# Metabolic effects of orally administered small-molecule agonists of GPR55 and GPR119 in multiple low-dose streptozotocin-induced diabetic and incretin-receptor-knockout mice

**DOI:** 10.1007/s00125-016-4108-z

**Published:** 2016-09-27

**Authors:** Aine M. McKillop, Brian M. Moran, Yasser H. A. Abdel-Wahab, Noella M. Gormley, Peter R. Flatt

**Affiliations:** School of Biomedical Sciences, Ulster University, Cromore Road, Coleraine, BT52 1SA Northern Ireland UK

**Keywords:** Beta cell regeneration, Diabetes, Fatty acid agonists, Glucose homeostasis, G-protein-coupled receptors, Insulin secretion, Multiple low-dose streptozotocin

## Abstract

**Aims/hypothesis:**

Abnormal cannabidiol (Abn-CBD) and AS-1269574 are potent selective agonists for GPR55 and GPR119, respectively. The present study evaluated the actions and ability of these small-molecule agonists to counteract experimental diabetes in mice.

**Methods:**

Diabetes was induced in NIH Swiss mice by five consecutive daily intraperitoneal injections of 40 mg/(kg body weight) streptozotocin. Diabetic mice received daily oral administration of Abn-CBD or AS-1269574 (0.1 μmol/kg) or saline vehicle (0.9% wt/vol. NaCl) over 28 days. Body weight, food intake, fluid intake, plasma glucose, insulin, glucose tolerance, insulin release, lipid profile and pancreatic morphology were examined. Mechanism of action of agonists was assessed in acute studies using incretin-receptor-knockout mice.

**Results:**

Abn-CBD and AS-1269574 decreased plasma glucose (20–26%, *p* < 0.05) and increased circulating insulin (47–48%, *p* < 0.05) by 10–28 days, compared with saline-treated diabetic controls. Food intake and polydipsia were reduced by both agonists (21–23%, *p* < 0.05 and 33–35%, *p* < 0.01, respectively). After 28 days of treatment, plasma glucagon concentrations were reduced (*p* < 0.01) and glucose tolerance was enhanced by 19–44% by Abn-CBD (*p* < 0.05 or *p* < 0.001) and AS-1269574 (*p* < 0.05 to *p* < 0.001). Plasma insulin responses were improved (*p* < 0.01) and insulin resistance was decreased (*p* < 0.05 or *p* < 0.01) in both Abn-CBD- and AS-1269574-treated groups. Triacylglycerols were decreased by 19% with Abn-CBD (*p* < 0.05) and 32% with AS-1269574 (*p* < 0.01) while total cholesterol was reduced by 17% (*p* < 0.01) and 15% (*p* < 0.05), respectively. Both agonists enhanced beta cell proliferation (*p* < 0.001) although islet area was unchanged. Acute studies in *Gipr*- and *Glp1r*-knockout mice revealed an important role for the glucagon-like peptide 1 (GLP-1) receptor in the actions of both agonists, with the glucose-lowering effects of Abn-CBD also partly mediated through the glucose-dependent insulinotropic peptide (GIP) receptor.

**Conclusions/interpretation:**

These data highlight the potential for fatty acid G-protein-coupled receptor-based therapies as novel insulinotropic and glucose-lowering agents acting partly through the activation of incretin receptors.

**Electronic supplementary material:**

The online version of this article (doi:10.1007/s00125-016-4108-z) contains peer-reviewed but unedited supplementary material, which is available to authorised users.

## Introduction

NEFAs play a complex role in glucose homeostasis and the pathogenesis of type 2 diabetes [1] and recent studies have shown that G-protein-coupled receptors (GPCRs) are involved in sensing NEFAs [2, 3]. NEFAs have an emerging role in improving glucose-stimulated insulin release from pancreatic beta cells and improving insulin sensitivity in liver and skeletal muscle [4]. GPCRs have different affinities for fatty acids of varying chain lengths. Thus whereas medium- and long-chain fatty acids activate GPR40 and GPR120, short-chain fatty acids are known to serve as ligands for GPR41 and GPR43 [4]. Furthermore, novel receptors GPR55 and GPR119 have recently been shown to affect blood glucose control, and activation of these receptors with novel synthetic fatty acids may have therapeutic potential for type 2 diabetes [5].

GPR119 has been identified on pancreatic beta cells and intestinal L cells and K cells and has the ability to enhance glucose-stimulated insulin release and the secretion of both glucagon-like peptide-1 (GLP-1) and glucose-dependent insulinotropic peptide (GIP) [6]. These incretin hormones have a variety of actions in pancreatic islets including enhancement of insulin biosynthesis and glucose-stimulated insulin release, stimulation of beta cell proliferation and inhibition of beta cell apoptosis [7]. GLP-1 additionally inhibits glucagon secretion [8]. GPR119 has been shown to bind a variety of lipid-derived ligands, as well as a range of small synthetic molecules. Several endogenous and synthetic GPR119 agonists have been shown to exhibit insulin-secretory properties in clonal beta cells, isolated islets and in vivo in mice [9, 10]. AS-1269574 is a specific and potent fatty acid GPR119 agonist that enhances glucose-dependent insulin secretion [9, 10]. AS-1269574 has also been implicated in GLP-1 secretion and increased proglucagon gene promoter activity via GPR119 in a mouse L cell line (GLUTag) [11]. A recent study found that a synthetic GPR119 agonist (PSN-632408) can enhance beta cell regeneration, improve islet cell graft survival and augment plasma active GLP-1 [12]. Previous reports have found that GPR119 agonists exhibit enhanced potency in vivo in combination with inhibition of enzymes such as fatty acid amide hydrolase inhibitor (URB-597) [13] or dipeptidyl-peptidase-IV (DPP-IV; sitagliptin) [14, 15].

GPR55 is expressed in the central nervous system, ileum, adipose tissue and endocrine pancreas, being predominantly on insulin-secreting beta cells [16, 17]. GPR55 was initially de-orphanised as a cannabinoid receptor and this receptor binds many cannabinoid compounds. However, some GPR55 ligands are not cannabinoids and do not bind to either cannabinoid receptor 1 (CB1) or 2 (CB2) [16]. Cannabidiol (CBD) is a known GPR55 antagonist and has structural similarity to cannabinol and Δ^9^-tetrahydrocannabinol [18]. A range of synthetic CBD analogues have been synthesised, including abnormal cannabidiol (Abn-CBD), O-1918 and O-1602, which all act via GPR55 [5]. GPR55 has been associated with several physiological roles including anti-inflammatory activity [19], osteoclast function [20], insulin secretion and glucose homeostasis [17, 21, 22]. GPR55 agonists exhibited insulinotropic properties in clonal beta cells, isolated islets and in vivo in mice [17, 21, 22], with Abn-CBD noted as having the maximum potency and selectivity for GPR55 [21]. A recent study also using mice found that ablation of the GPR55 receptor increases adiposity and insulin resistance, selectively decreasing physical activity [23]. Further studies are required to assess the efficacy of Abn-CBD administration in the treatment of diabetes and other obesity-related diseases.

Recently we identified Abn-CBD and AS-1269574 as potent selective agonists for GPR55 and GPR119, respectively; both agonists exhibited acute glucose-lowering and insulinotropic properties in mice [9, 21]. In the present study we have evaluated the long-term glucose-lowering effects of small-molecule agonists Abn-CBD and AS-1269574 in mice with diabetes induced by multiple-low-dose streptozotocin (STZ). To gain further information on the potential mechanism of action of these GPCR agonists, glucose-lowering and insulin-releasing properties were assessed in vivo using *Gipr*- and *Glp1r*-knockout mice.

## Methods

See electronic supplementary material (ESM) Methods for details of materials.

### Animals

Male NIH Swiss mice (10–16 weeks old) were purchased from Harlan, UK. *Gipr*-and *Glp1r*-knockout mice crossed with the C57BL/6 strain (courtesy of B. Thorens, Lausanne, Switzerland and D. J. Drucker, Toronto, ON, Canada) and age-matched control wild-type C57BL/6 mice were obtained from an in-house breeding colony. The study was conducted in accordance with the Guide for the Care and Use of Laboratory Animals (2011) and the UK Animal (Scientific Procedures) Act 1986 and ARRIVE guidelines for reporting experiments involving animals [24].

### Chronic administration of Abn-CBD and AS-1269574 in STZ-induced diabetic mice

In a long-term study (28 days), the effects of daily oral administration of Abn-CBD and AS-1265974 (both at 0.1 μmol/[kg body weight]) [9, 21] or saline vehicle (0.9% wt/vol. NaCl) were examined in multiple-low-dose streptozotocin-induced diabetic NIH Swiss mice. To induce diabetes, NIH Swiss mice fasted for 4 h received five consecutive daily intraperitoneal injections of STZ (40 mg/[kg body weight]). See ESM Methods for further details.

### Acute in vivo effects of Abn-CBD and AS-1269574 in incretin-receptor-knockout mice

Age-matched, non-fasted *Gipr*-knockout, *Glp1r*-knockout and wild-type C57/BL6 mice (*n* = 6) received an oral injection of glucose alone (18 mmol/[kg body weight]) or in combination with either Abn-CBD or AS-1269574 (both at 0.1 μmol/[kg body weight]). See ESM Methods for further details.

### Acute in vivo effects of Abn-CBD and AS-1269574 and relevant antagonists in lean mice

Fasted male lean NIH Swiss mice received glucose (18 mmol/[kg body weight]) alone or in combination with GPR119 agonist AS1269754 and GPR119 antagonist exendin (9-39) (0.1 μmol/[kg body weight]) or GPR55 agonist Abn-CBD and GPR55 antagonist CBD orally or intraperitoneally and glucose/insulin was measured.

### Histology

Pancreatic tissues were removed at 28 days and processed as previously reported [21]. See ESM Methods for further details.

### Biochemical analysis

Analysis of blood samples was undertaken as previously reported [21, 25]. See ESM Methods for further details.

### Statistics

Data are expressed as the means ± SEM. Results were compared using the Student’s *t* test or one-way ANOVA on Prism graph pad version 5.0. Differences in data were considered to be statistically significant for *p* < 0.05.

## Results

### Effect of Abn-CBD and AS-1269574 on food intake, fluid intake, body weight, non-fasting plasma glucose, insulin, glucagon and pancreatic insulin content

**Fig. 1:**
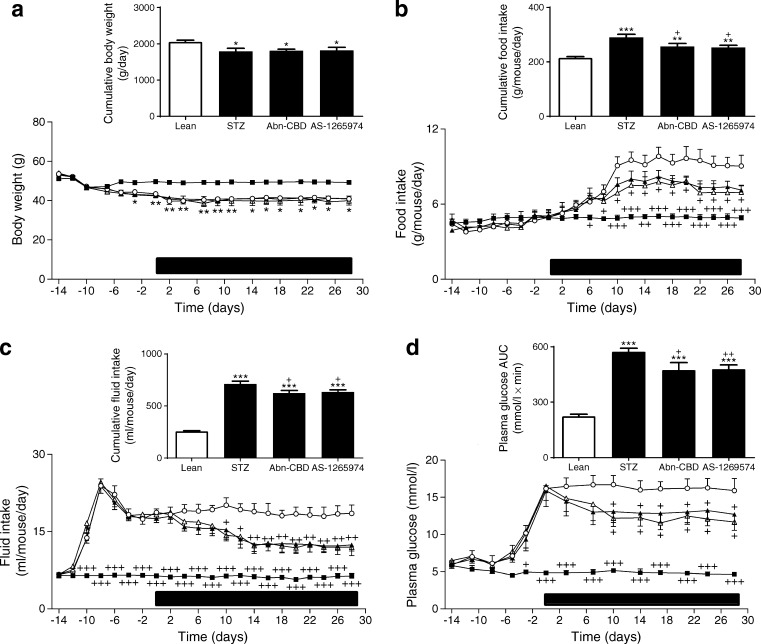
Effect of daily administration of Abn-CBD and AS-1269574 on body weight (**a**), food intake (**b**), fluid intake (**c**) and plasma glucose (**d**) in STZ-induced diabetic mice. Variables were measured before and during 28 day treatment with Abn-CBD, AS-1269574 or saline vehicle (treatment period indicated by the horizontal black bar). Black squares, non-diabetic mice treated with saline (normal); white circles, STZ-induced diabetic mice treated with saline vehicle; black triangles, diabetic mice treated with Abn-CBD; white triangles, diabetic mice treated with AS-1269574. Values are means ± SEM for six mice. *p < 0.05, **p < 0.01 and ***p < 0.001 compared with normal mice; ^+^ *p* < 0.05, ^++^ *p* < 0.01 and ^+++^ *p* < 0.001 compared with diabetic mice

**Fig. 2:**
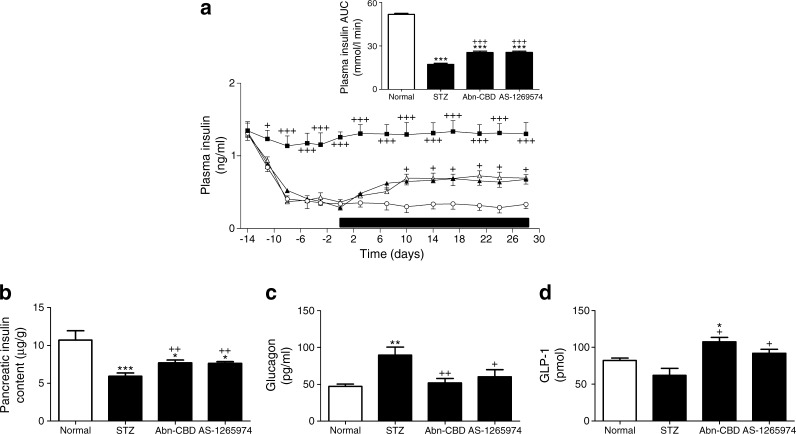
Effect of daily administration of Abn-CBD and AS-1269574 on non-fasting plasma insulin (**a**), pancreatic insulin content (**b**), plasma glucagon (**c**) and GLP-1 (**d**) in STZ-induced diabetic mice. In (**a**) variables were measured before and during 28 days of treatment with Abn-CBD, AS-1269574 or saline vehicle (treatment period indicated by the horizontal black bar) and in (**b**–**d**) they were measured after 28 days of treatment. Black squares, non-diabetic mice treated with saline (normal); white circles, STZ-induced diabetic mice treated with saline vehicle; black triangles, diabetic mice treated with Abn-CBD; white triangles, diabetic mice treated with AS-1269574. Values are means ± SEM for six mice. *p < 0.05, **p < 0.01 and ***p < 0.001 compared with normal mice; ^+^ *p* < 0.05, ^++^ *p* < 0.01 and ^+++^ *p* < 0.001 compared with diabetic mice

Multiple-low-dose STZ lowered body weight by 10% (*p* < 0.05) (Fig. 1a). Administration of the GPCR agonists Abn-CBD and AS-1269574 over 28 days had no effect on body weight. Food intake was decreased by 21–23% by Abn-CBD (*p* < 0.05) and AS-1265974 (*p* < 0.05) (Fig. 1b). Non-fasting plasma glucose was improved by Abn-CBD (20%, *p* < 0.05) and AS-1269574 (26%, *p* < 0.05) (Fig. 1d) while polydipsia was decreased by 33–35% with Abn-CBD (*p* < 0.01) and AS-1269574 (*p* < 0.01) (Fig. 1c). Non-fasting plasma insulin was increased by Abn-CBD (47%, *p* < 0.05) and AS-1269574 (48%, *p* < 0.05) by 10–28 days (Fig. 2a). This was associated with increased pancreatic insulin content in both groups treated with synthetic agonists (*p* < 0.01) (Fig. 2b). Plasma glucagon was decreased (*p* < 0.05 or *p* < 0.01) and GLP-1 was increased (*p* < 0.05) in non-fasting terminal plasma by both Abn-CBD and AS-1269574, compared with measurements in STZ-treated control mice (Fig. 2c, d).

### Effect of Abn-CBD and AS-1269574 on oral glucose tolerance and insulin sensitivity

**Fig. 3:**
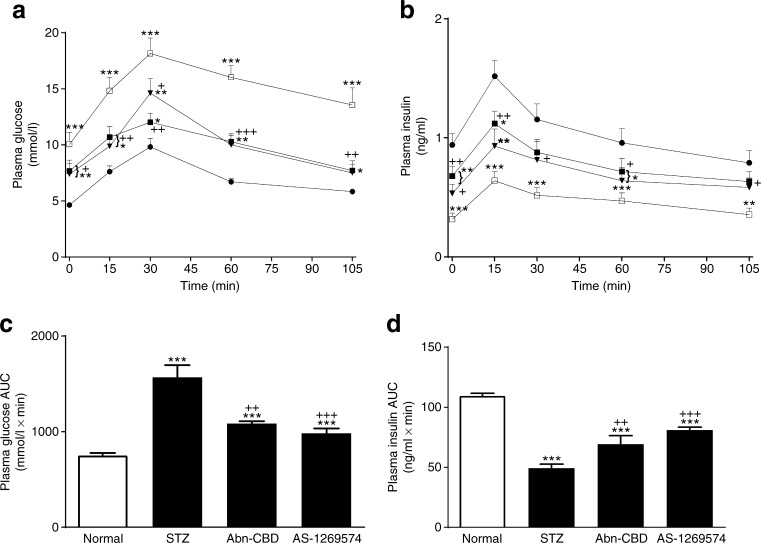
(**a**, **b**) Effect of daily administration of Abn-CBD and AS-1269574 on glucose tolerance (**a**) and plasma insulin response to glucose (**b**) in STZ-induced diabetic mice fasted for 18 h. OGTT (18 mmol/[kg body weight]) was conducted 28 days after treatment with Abn-CBD, AS-1269574 or saline vehicle. (**c**, **d**) Plasma glucose (**c**) and insulin (**d**) AUC values for 0–105 min post injection are shown. Black circles, non-diabetic mice treated with saline (normal); white squares, STZ-induced diabetic mice treated with saline vehicle; black triangles, diabetic mice treated with Abn-CBD; black squares, diabetic mice treated with AS-1269574. Values are means ± SEM for six mice. *p < 0.05, **p < 0.01 and ***p < 0.001 compared with normal mice; ^+^ *p* < 0.05, ^++^ *p* < 0.01 and ^+++^ *p* < 0.001 compared with diabetic mice

**Fig. 4:**
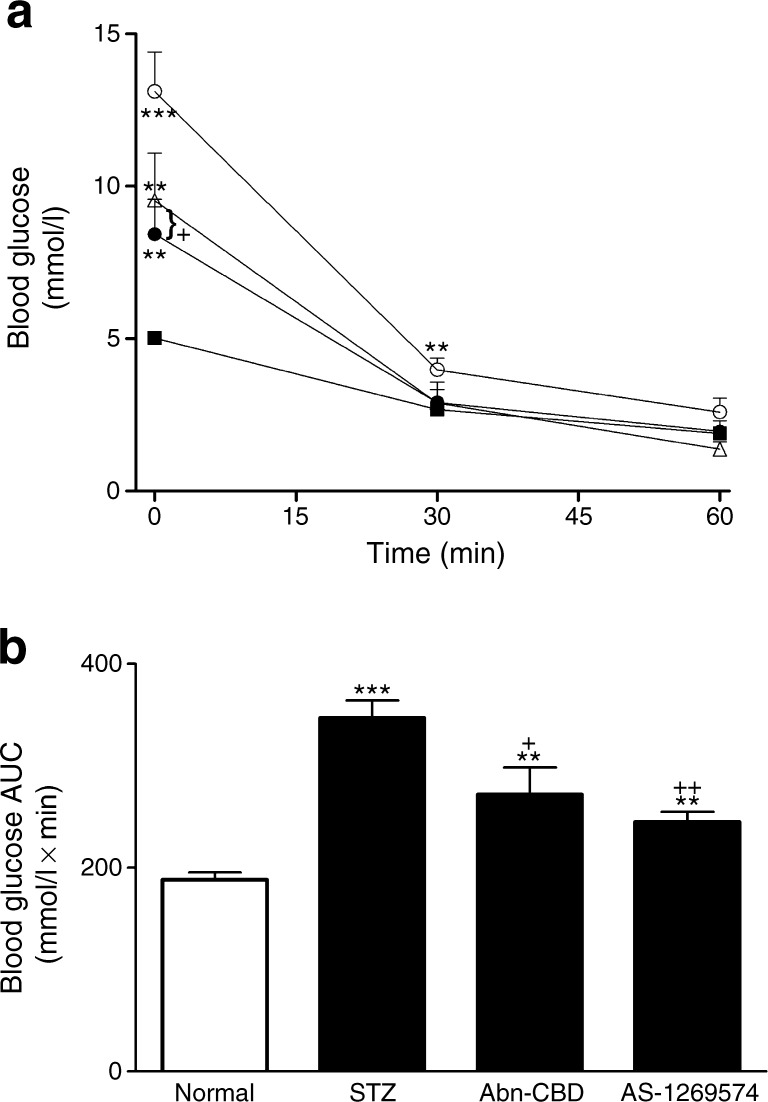
Effect of daily administration of Abn-CBD and AS-1269574 on insulin sensitivity depicted in terms of glucose (**a**) and AUC for glucose values (**b**) in STZ-induced diabetic mice. Insulin sensitivity tests (25 U/[kg body weight]) were conducted after 28 days of treatment with Abn-CBD and AS-1269574 or saline vehicle. Black squares, non-diabetic mice treated with saline (normal); white circles, STZ-induced diabetic mice treated with saline; white triangles, diabetic mice treated with Abn-CBD; black circles, diabetic mice treated with AS-1269574. Values are means ± SEM for six mice. **p < 0.01 and ***p < 0.001 compared with normal mice; ^+^ *p* < 0.05 and ^++^ *p* < 0.01 compared with diabetic mice

Daily oral administration of GPCR agonists for 28 days decreased the glycaemic excursion during an OGTT by 19–44% (Abn-CBD, *p* < 0.05 or *p* < 0.001) and 23–43% (AS-1269574, *p* < 0.05 or *p* < 0.001) compared with the control STZ group (Fig. 3a, c). In addition, Abn-CBD and AS-1269574 augmented glucose-stimulated insulin by 27–40% (*p* < 0.05 or *p* < 0.01) and 35–53% (*p* < 0.05 or *p* < 0.01), respectively (Fig. 3b, d). Plasma glucose concentrations and AUC values following injection of exogenous insulin were also reduced by Abn-CBD (*p* < 0.05) and AS-1269574 (*p* < 0.01) after 28 days of treatment (Fig. 4).

### Effect of Abn-CBD and AS-1269574 on dual-energy x-ray absorption measurements and non-fasting plasma lipid profile

**Fig. 5:**
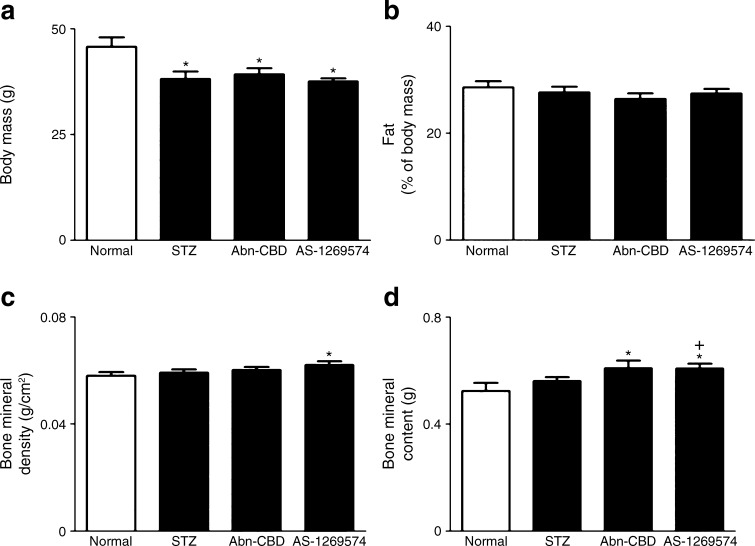
Effect of daily administration of Abn-CBD and AS-1269574 on total body mass (**a**), fat (**b**), bone mineral density (**c**) and bone mineral content (**d**) as measured by DEXA scanning in STZ-induced diabetic mice. Variables were measured after 28 days of treatment with Abn-CBD, AS-1269574 or saline vehicle. Values are means ± SEM for six mice. *p < 0.05 compared with non-diabetic mice treated with saline; ^+^ *p* < 0.05 compared with diabetic mice

**Fig. 6:**
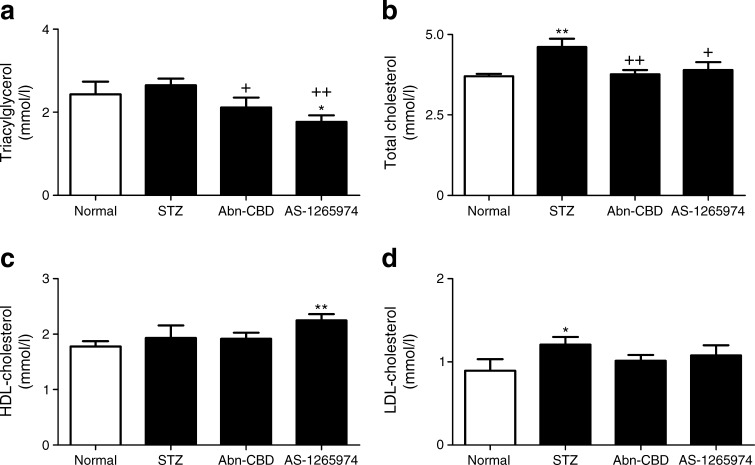
Effect of daily administration of Abn-CBD and AS-1269574 on plasma triacylglycerols (**a**), total cholesterol (**b**), HDL-cholesterol (**c**) and LDL-cholesterol (**d**) in STZ-induced diabetic mice. LDL-cholesterol was calculated using the Friedewald equation (LDL-cholesterol = total cholesterol minus HDL-cholesterol minus [triacylglycerols/2.2]). Variables were measured after 28 days of treatment with Abn-CBD, AS-1269574 or saline vehicle. Values are means ± SEM for six mice. *p < 0.05 and **p < 0.01 compared with non-diabetic mice treated with saline; ^+^ *p* < 0.05 and ^++^ *p* < 0.01 compared with diabetic mice

Dual-energy x-ray absorption (DEXA) scanning revealed a decreased body mass in the three groups of STZ-treated mice (*p* < 0.05) (Fig. 5a), with no effects on body fat expressed as percentage of body mass (Fig. 5b). AS-1269574 and Abn-CBD had no effect on body weight or fat content, compared with STZ-treated controls (Fig. 5a, b). GPR119 agonist AS-1269574 enhanced both bone mineral density (6%, *p* < 0.05) and bone mineral content (13%, *p* < 0.05), while GPR55 agonist Abn-CBD augmented bone mineral content (14%, *p* < 0.05) (Fig. 5c, d). After 28 days of treatment, non-fasting triacylglycerols decreased by 19% with Abn-CBD (*p* < 0.05) and 32% with AS-1269574 (*p* < 0.01) compared with diabetic controls (Fig. 6a). Similarly, total cholesterol was reduced by both Abn-CBD (17%, *p* < 0.01) and AS-1269574 (15%, *p* < 0.05) (Fig. 6b), while AS-1269574 augmented HDL-cholesterol concentrations by 19% (*p* < 0.01) when compared to the lean group (Fig. 6c).

### Effect of Abn-CBD and AS-1269574 on islet morphology

**Fig. 7:**
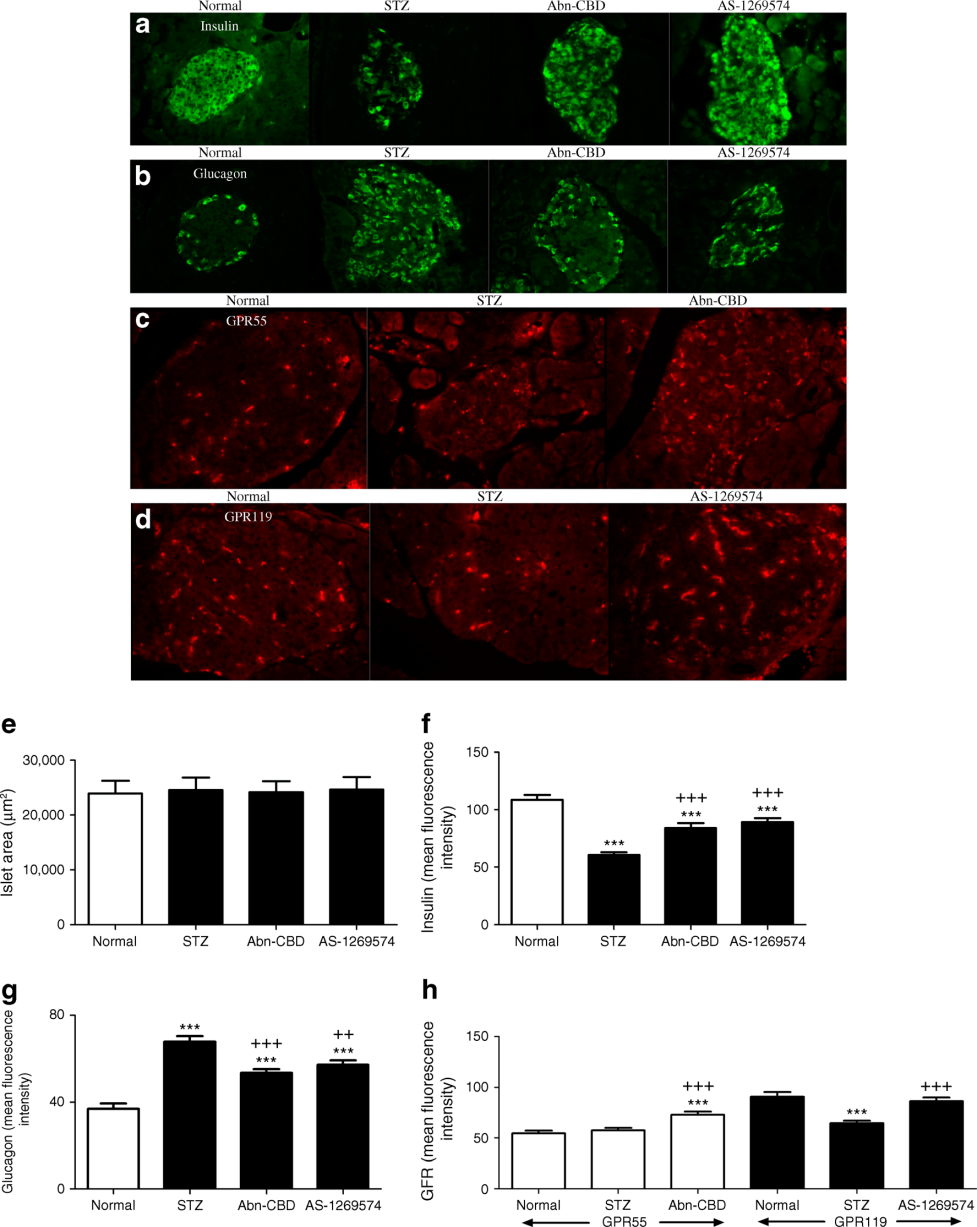
(**a**–**d**) Representative images of immunocytochemical staining for insulin (**a**), glucagon (**b**), GPR55 (**c**) and GPR119 (**d**) in the pancreas of STZ-induced diabetic mice after 28 days of treatment with Abn-CBD, AS-1269574 or saline vehicle. Magnification × 40. (**e**–**h**) Islet area (**e**) and relative fluorescence for insulin (**f**), glucagon (**g**), GPR55 and GPR119 (**h**) determined using cell F software. Values are means ± SEM for six mice, 25 islets per group. ***p < 0.001 compared with non-diabetic mice treated with saline; ^++^ *p* < 0.01 and ^+++^ *p* < 0.001 compared with diabetic mice

Multiple-low-dose STZ-induced diabetes was associated with diminished insulin-positive beta cells (*p* < 0.001; Fig. 7a, f), augmented glucagon-positive alpha cells (*p* < 0.001; Fig. 7b, g) and decreased GPR119 expression (*p* < 0.001; Fig. 7d, h). These islet abnormalities were partially reversed in Abn-CBD- and AS-1269574 treated groups, with increased insulin-positive beta cells (*p* < 0.001; Fig. 7a, f) and reduced glucagon expression in alpha cells (*p* < 0.01 or *p* < 0.001; Fig. 7b, g) when comparing the Abn-CBD- and AS-1269574-treated mice with saline-treated STZ-induced diabetic mice. Additionally, administration of Abn-CBD enhanced GPR55 islet expression (*p* < 0.001; Fig. 7c, h) while AS-1269574 increased GPR119 expression (*p* < 0.001; Fig. 7d, h), compared with saline-treated STZ controls.

**Fig. 8:**
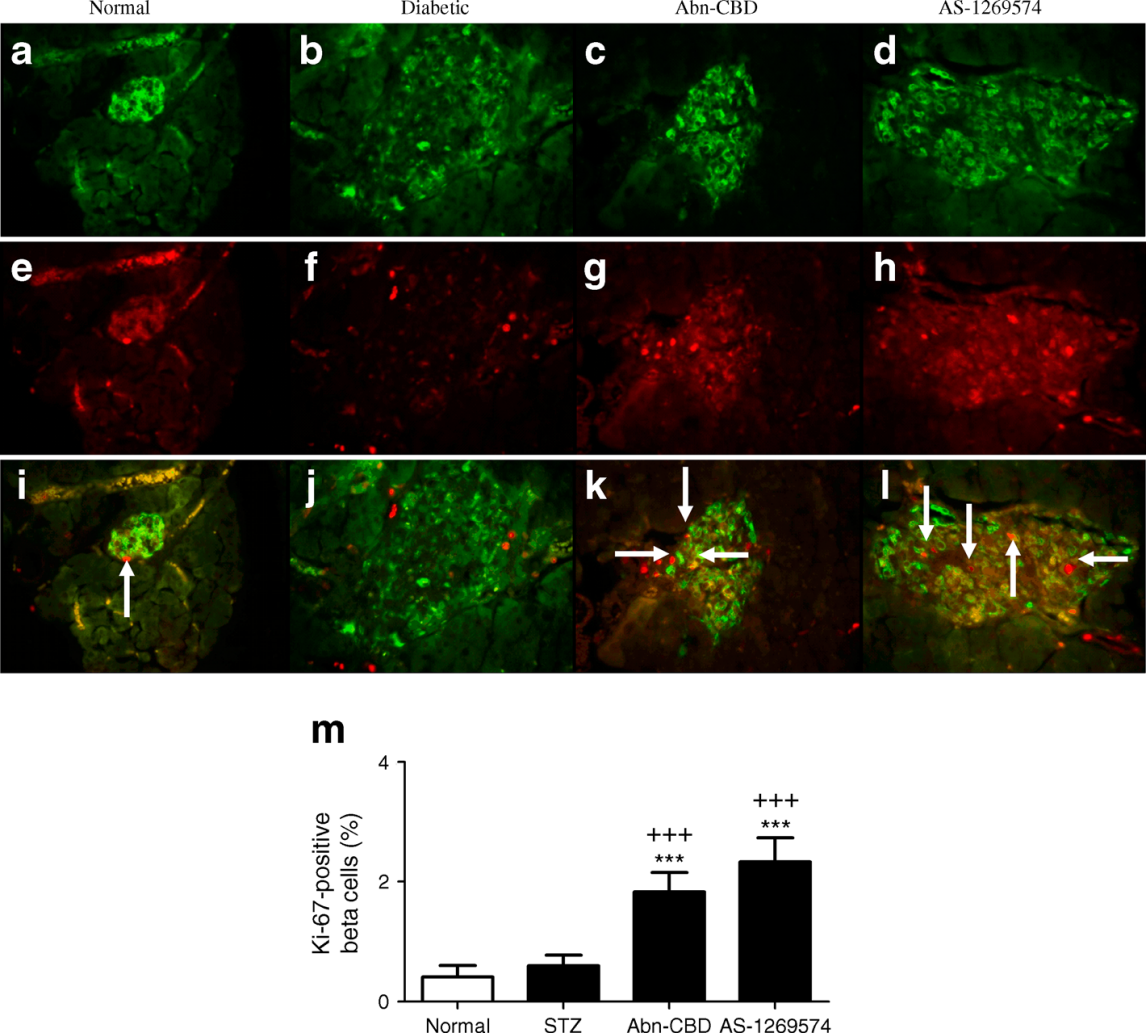
Immunocytochemical staining for insulin and Ki-67 in the pancreas of STZ-induced diabetic mice after 28 days of treatment with Abn-CBD, AS-1269574 or saline vehicle. (**a**–**l**) Representative images are shown for insulin (**a**–**d**), Ki-67 (**e**–**h**) and merged insulin and Ki-67 (**i**–**l**) Magnification × 40. (**m**) Percentage of Ki-67-positive beta cells. Values are means ± SEM for six mice, 25 islets per group. ***p < 0.001 compared with non-diabetic mice treated with saline; ^+++^ *p* < 0.001 compared with diabetic mice. Arrows indicate insulin/Ki-67 co-positive beta cells

To investigate beta cell proliferation with GPR119 and GPR55 agonists, Ki-67 was used as a nuclear marker for cellular replication (Fig. 8). Both Abn-CBD and AS-1269574 enhanced the percentage of insulin/Ki-67 co-positive cells (Fig. 8k–l). Non-diabetic mice treated with saline (0.4%) and untreated STZ-induced diabetic (0.6%) mice exhibited minimal beta cell proliferation while insulin/Ki-67 positive cell counts were significantly enhanced in Abn-CBD (1.8%, *p* < 0.001) and AS-1269574 (2.3%, *p* < 0.001) treatment groups (Fig. 8m).

### Acute effect of Abn-CBD and AS-1269574 in wild-type C57BL/6 mice and in *Gipr*- and *Glp1r*-knockout mice

**Fig. 9:**
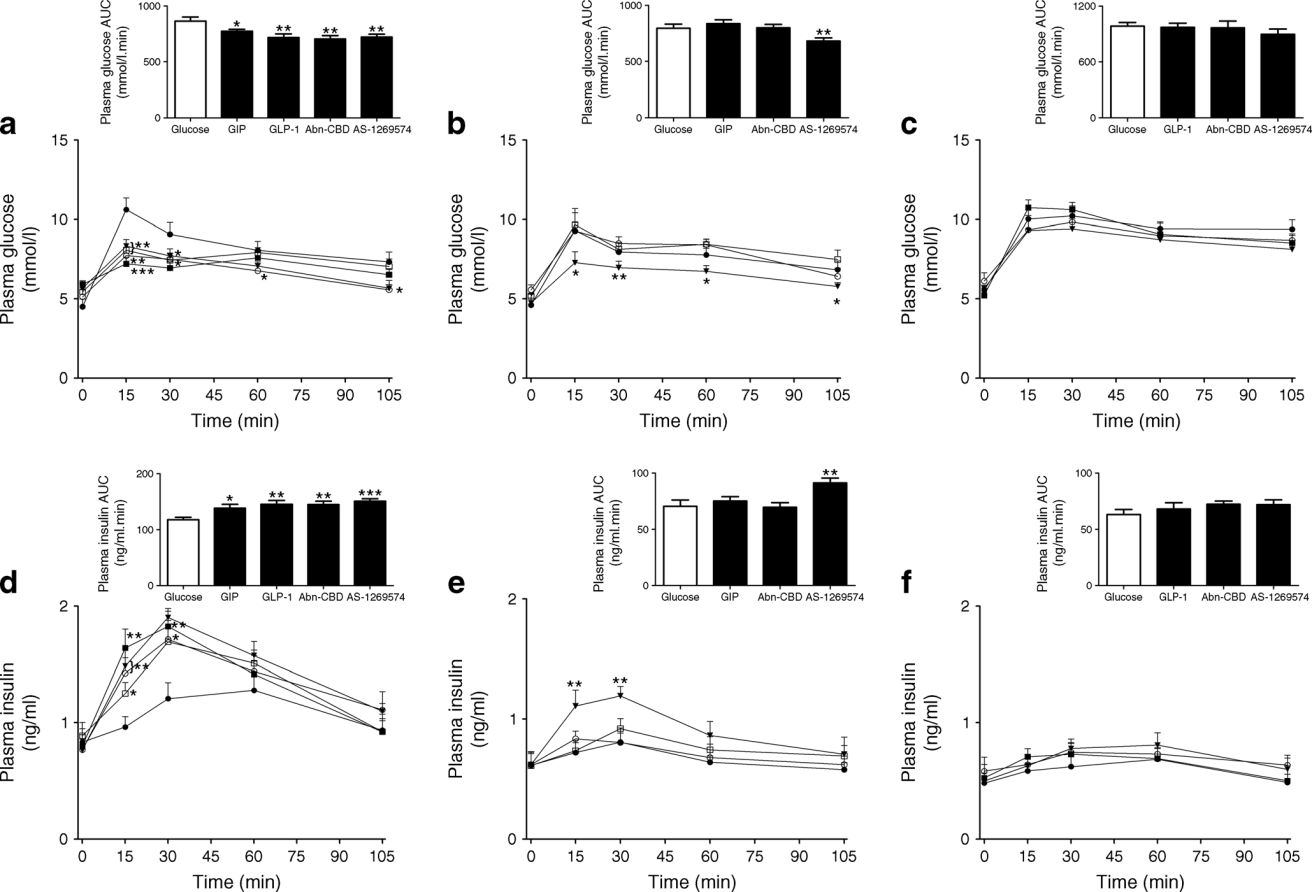
Acute effect of Abn-CBD and AS-1269574 on glucose tolerance, and the plasma insulin response to oral glucose in C57BL/6 wild-type mice (**a**, **d**) and in C57BL/6 mice with knockout of *Gip* (**b**, **e**) or *Glp1* receptors (**c**, **f**). Plasma glucose (**a**–**c**) and insulin (**d**–**f**) were determined before and after oral administration of glucose (18 mmol/[kg body weight]) alone or in combination with Abn-CBD or AS-1269574 (0.1 μmol/[kg body weight]). Black circles, glucose; white squares, glucose + GIP (25 nmol l^−1^ kg^−1^); black squares, glucose + GLP-1 (25 nmol l^−1^ kg^−1^); white circles, glucose + Abn-CBD (0.1 μmol l^−1^ kg^−1^); black triangles, glucose + AS-1269574 (0.1 μmol l^−1^ kg^−1^). Values are means ± SEM for six mice. *p < 0.05, **p < 0.01 and ***p < 0.001 compared with 18 mmol/l glucose

Administration of GIP or GLP-1 had no biological effects in either *Gipr*-knockout mice or *Glp1r*-knockout mice, respectively (Fig. 9b, c, e and f). In contrast, in C57BL/6 mice GIP lowered plasma glucose by 18–24% (*p* < 0.05 or *p* < 0.01) and enhanced glucose-stimulated insulin release by 26–29% (*p* < 0.05) (Fig. 9a, d). Similarly, GLP-1 attenuated glucose levels by 23–32% (*p* < 0.05 or *p* < 0.001) and stimulated insulinotropic ability by 33–40% (*p* < 0.01) in wild-type controls (Fig. 9a, d). In these control mice, GPR55 agonist Abn-CBD decreased glucose by 16–27% (*p* < 0.05 or *p* < 0.01) and increased insulin secretion by 29–31% (*p* < 0.01) (Fig. 9a, d). GPR119 agonist AS-1269574 had a similar effect, reducing glucose by 15–22% (*p* < 0.05 or *p* < 0.01) and augmenting glucose-stimulated insulin release by 35–37% (*p* < 0.01) (Fig. 9a, d).

In *Gipr*-knockout mice, the glucose-lowering and insulinotropic actions of Abn-CBD were diminished while AS-1269574 decreased the glycaemic excursion by 14–24% (*p* < 0.05 or *p* < 0.01) and enhanced glucose-induced insulin secretion by 33–35% (*p* < 0.01) (Fig. 9b, e). Both Abn-CBD and AS-1269574 had diminished glucose-lowering and insulinotropic effects in *Glp1r*-knockout mice (Fig. 9c, f).

### Acute effect of GPR119 and GPR55 agonists in the presence of antagonists

**Fig. 10:**
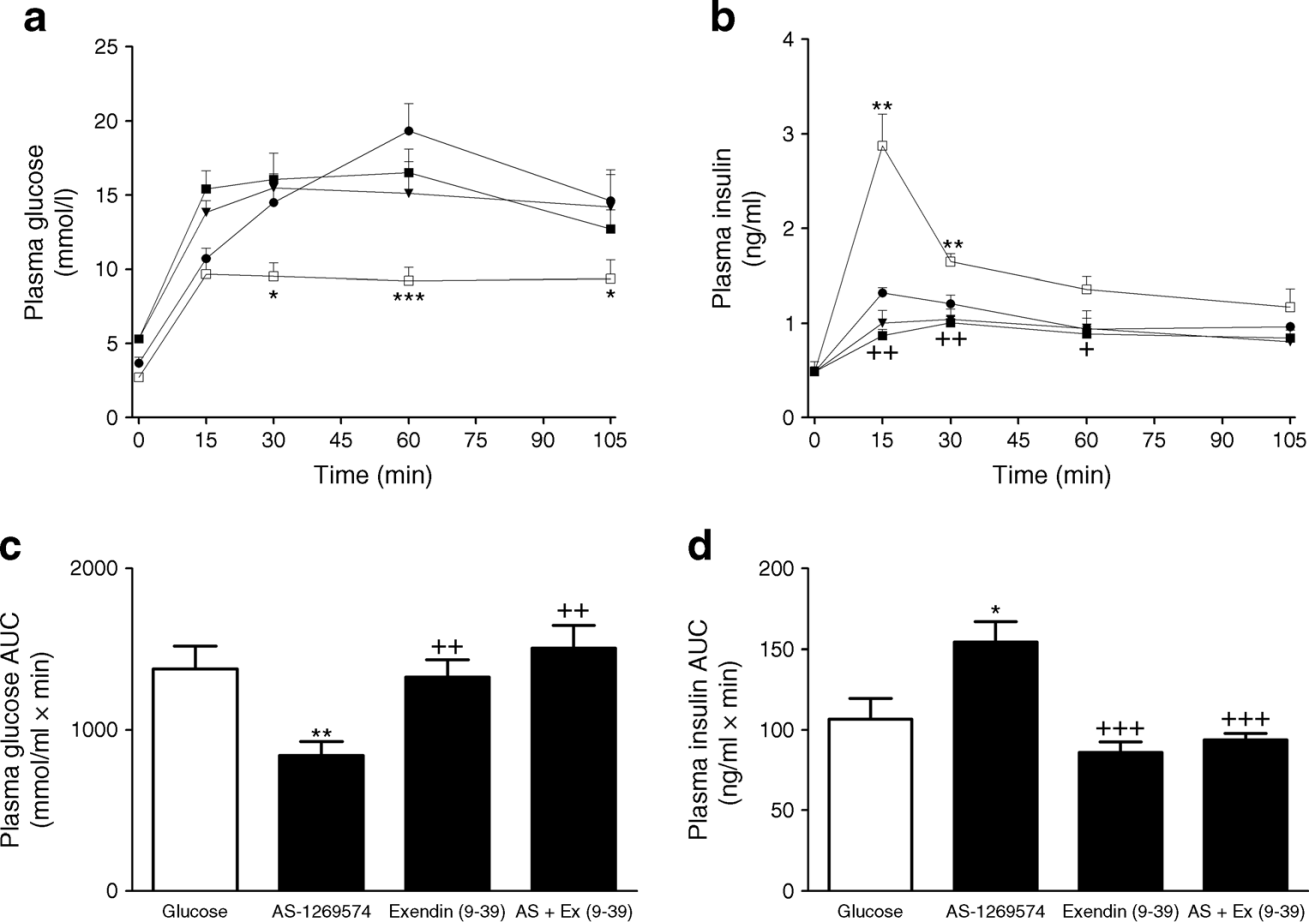
Acute effect of GPR119 agonist in combination with GPR119 antagonist exendin (9-39) on plasma glucose (**a**, **c**) and plasma insulin (**b**, **d**). Fasted male lean NIH Swiss mice (*n* = 6) received glucose (18 mmol/[kg body weight]) alone or in combination with GPR119 agonist AS1269754 and/or GPR119 antagonist exendin (9-39) (0.1 μmol/[kg body weight]). Values are means ± SEM. Black circles, glucose alone; white squares, glucose + AS-1269574; black triangles, glucose + exendin (9-39); black squares, glucose + AS-1269574 + exendin (9-39). *p < 0.05, **p < 0.01 and ***p < 0.001 compared with glucose; ^+^ *p* < 0.05, ^++^ *p* < 0.01 and ^+++^ *p* < 0.001 compared with glucose + AS-1269574

**Fig. 11:**
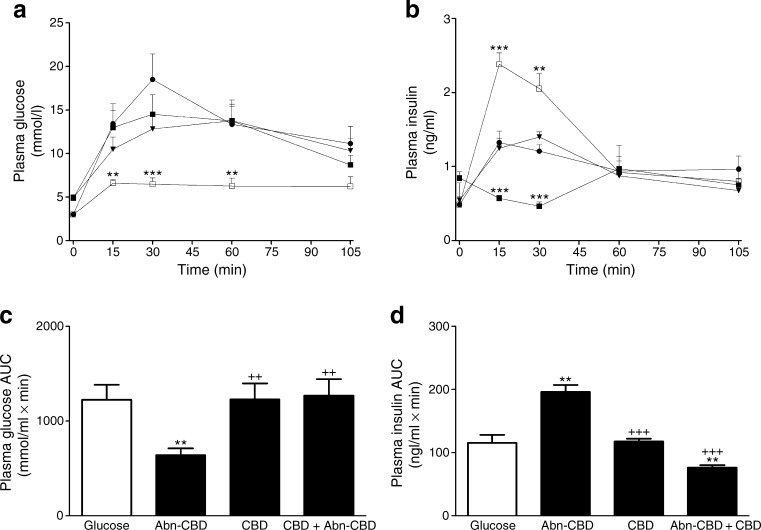
Acute effect of GPR55 agonist in combination with GPR55 antagonist CBD on plasma glucose (**a**, **c**) and plasma insulin (**b**, **d**). Fasted male lean NIH Swiss mice (*n* = 6) received glucose (18 mmol/[kg body weight]) alone or in combination with GPR55 agonist Abn-CBD and/or GPR55 antagonist CBD. Black circles, glucose alone; white squares, glucose + Abn-CBD; black triangles, glucose + CBD; black squares, glucose + Abn-CBD + CBD. Values are means ± SEM. **p < 0.01 and ***p < 0.001 compared with glucose; ^++^ *p* < 0.01 and ^+++^ *p* < 0.001 compared with glucose + Abn-CBD

AS-1269574 administered in combination with antagonist exendin (9-39) resulted in a 44% increase in blood glucose (*p* < 0.01) (Fig. 10) while insulin release was reduced by 39% (*p* < 0.001) (Fig. 10) when compared with AS-1269574 alone. In the presence of GPR55 antagonist CBD and Abn-CBD, glucose increased 49% (*p* < 0.01), compared with levels noted with agonist alone (Fig. 11). Insulin levels were reduced by 61% (*p* < 0.001) using AUC data (Fig. 11), when Abn-CBD was administered in combination with GPR55 antagonist CBD.

## Discussion

Much interest has been focused recently on fatty acid GPCRs in relation to their potential beneficial effects on glucose homeostasis in type 2 diabetes and other obesity-related diseases [1–3, 26]. Several novel fatty acid receptors, including GPR55 and GPR119, have shown promise as emerging targets of diabetes therapies. Previous research has highlighted the potent acute glucose-lowering and insulinotropic ability of the GPCR agonists Abn-CBD and AS-1269574 [9, 21] and our acute in vivo studies, using GPR55 and GPR119 antagonists, have confirmed the specificity of Abn-CBD and AS-1269574 in islets.

The present study evaluated the glucose-lowering and insulinotropic properties of small-molecule agonists Abn-CBD and AS-1269574 in multiple-low-dose STZ-induced diabetes in mice. This is a commonly employed means of inducing diabetes [27, 28], which in the present study was associated with decreased body weight, hyperphagia, polydipsia, moderate hyperglycaemia, hyperglucagonaemia and hypoinsulinaemia but with good numbers of surviving functional beta cells, representing a model of mild type 1 diabetes. Daily oral administration of Abn-CBD and AS-1269574 lowered plasma glucose, plasma glucagon and food and fluid intake. In harmony with this, plasma insulin and pancreatic insulin content were increased in both treatment groups. Following long-term administration of the agonists, glucose tolerance and insulin sensitivity were markedly improved. Additionally, total cholesterol and triacylglycerols were decreased by Abn-CBD and AS-1269574 and HDL-cholesterol was augmented by AS-1269574. Interestingly, our data would suggest a link between GPCR activation and suppression of hyperphagia. We found that GPR55 and GPR119 agonists decreased food consumption and appetite, though we observed no effect on overall body weight. This is in contrast to some published literature suggesting a link between GPR55 antagonism and reduced food consumption and body weight gain [22]. The accompanying decrease in fluid intake in the mice in our study most likely reflects the improved hyperglycaemic status following treatment with the two GPCR agonists. Moreover, GPR55 expression has been found to be increased in adipose tissue of obese individuals and further so in obese patients with type 2 diabetes, with GPR55 expression correlating with body weight, BMI and percentage fat mass [29]. Ex vivo studies using both adipose tissue explants and differentiated primary adipocytes show that l-α-lysophosphatidylinositol, an endogenous GPR55 agonist, increased the expression of genes stimulating fat deposition in adipose tissue and in differentiated adipocytes from visceral fat of obese patients it raised intracellular Ca^2+^ concentration. The GPR119 data is generally consistent with a recent study which found that AS-1907417, a modified form of AS-1269574, improved lipid profile, plasma insulin, plasma glucose and pancreatic insulin content over a 4 week treatment period in *db*/*db* mice [30]. These recent studies suggest that activation of GPR55 and GPR119 could play a role in weight management, energy load and lipid metabolism.

Very few previous studies have assessed long-term administration of AS-1269574 and Abn-CBD in animal models of diabetes. Interestingly, bone mineral content, assessed by DEXA, was increased by agonising GPR119 with AS-1269574. GPR55 is expressed on osteoclasts [20] and, in contrast to our observations, GPR55-receptor-knockout mice have been reported to exhibit enhanced quantity and thickness of trabecular bone [20]. This difference may reflect an adaptive response in mice deficient in GPR55. No data are currently available on GPR119 and bone metabolism. Of interest, recent studies have highlighted the importance of GIP [31] and GLP-1 receptors [32] in bone physiology, including bone strength and quality. This may explain the enhancement in bone mineral density and content by the GPCR agonists in this study as the receptors, in particular GPR119, has been implicated in GIP and GLP-1 secretion [6, 33, 34].

In the present study, multiple-low-dose STZ induced a relatively moderate form of diabetes associated with hypoinsulinaemia and mild effects on islet architecture. The islets contained good numbers of surviving positively stained insulin beta cells, increased numbers of glucagon-secreting alpha cells and decreased expression of the GPR119 receptor. Oral administration of Abn-CBD or AS-1269574 resulted in increased numbers of insulin-secreting beta cells and reduced hyperglucagonemia confirmed by decreased circulating plasma glucagon levels. The expression of GPR55 and GPR119 was enhanced by Abn-CBD and AS-1269574, respectively, with GPR119 reverting to normal expression levels. In addition, treatment with GPR55 and GPR119 agonists augmented beta cell proliferation and regeneration. Consistent with this, a recent study reported that the GPR119 agonist PSN-632408 increased GLP-1 secretion and beta cell proliferation as assessed by Ki-67 and BrdU staining; these effects were further augmented by DPP-IV inhibitor sitagliptin [14]. Many factors are known to play a role in the regulation of beta cell proliferation, including incretins, and further studies are required to investigate the possibility of parallel changes in apoptosis and to discover whether the increased cellular proliferation and replication observed in this study with long-term administration of GPCR agonists is a result of the action of incretins on beta cell proliferation and beta cell function. Both GIP and GLP-1 have been implicated in a number of protective functions in pancreatic beta cells including proliferation, neogenesis and anti-apoptosis [35].

This study has confirmed [6, 33, 34] that GPR119 agonists stimulate the release of GLP-1 from L cells. The GPR55 agonist Abn-CBD exhibited potent glucose-lowering and insulin-releasing properties in C57BL/6 wild-type mice, effects that were abolished in both *Gipr*- and *Glp1r*-knockout mice. The GPR119 agonist AS-1269574 displayed strong glucose-lowering and insulinotropic ability in wild-type C57BL/6 mice as well as *Gipr*-knockout mice. In contrast, GPR119 activation with AS-1269574 removed the glucose-lowering effect of the ligand in *Glp1r*-knockout mice. These data indicate that, in addition to direct stimulatory effects of the agonists on beta cells, the beneficial effects of Abn-CBD also involve the GIP and GLP-1 receptors and AS-1269574 activation of the GLP-1 receptor.

Overall these results indicate that GPCR small-molecule agonists Abn-CBD and AS-1269574 exert a broad spectrum of actions on glucose homeostasis, islet and enteroendocrine cells, lipid profiles and pancreatic and bone composition. Interestingly, few studies have assessed the role of GPR55 in diabetes [16, 18], with no literature probing the involvement of incretin hormones in the mechanism of action of GPR55. In contrast, several studies have identified the importance of GLP-1 [11, 12, 14, 15] and GIP [6] in GPR119 activation in glucose homeostasis. Similar to the findings of this study, the GPR119 agonist AS-1269574 has also been found to enhance plasma GLP-1 concentrations [11, 34]. Few studies have assessed the role of GPR119 in GIP signalling and the exact molecular mechanisms remain unclear. The synthetic agonist AR-231453 enhanced both GIP and GLP-1 levels in wild-type mice but not in *Gpr119*-knockout mice [34]. Additionally, AR-231453 stimulated GIP and GLP-1 release in wild-type mice and *Glp1r*- and *Gipr*-knockout mice [6]. Further studies are clearly warranted to assess the role of GPR119 and GPR55 agonists on the incretin pathway.

In conclusion, long-term administration of GPR55 agonist Abn-CBD and GPR119 agonist AS-1269574 improved glycaemic control in a multiple-low-dose STZ-induced mouse model of diabetes, resulting in decreased plasma glucose and glucagon and improved plasma insulin, GLP-1 and lipid profiles. Additionally, glucose tolerance and insulin sensitivity were enhanced, with positive actions on the pancreas and bone. The results also indicate an important action through the incretin pathway, suggesting that agonists capable of agonising both fatty acid GPCRs and incretin secretion may have therapeutic potential in the future.

## Electronic supplementary material

Below is the link to the electronic supplementary material.

ESM 1 (PDF 162 kb)

## Abbreviations

Abn-CBD: Abnormal cannabidiol
CB1: Cannabinoid receptor 1
CB2: Cannabinoid receptor 2
CBD: Cannabidiol
DEXA: Dual-energy x-ray absorption
DPP-IV: Dipeptidyl-peptidase-IV
GLP-1: Glucagon-like peptide-1
GIP: Glucose-dependent insulinotropic peptide
GPCR: G-protein coupled receptor
STZ: Streptozotocin
