# Supplementary material for: Metabolic effects of orally administered small-molecule agonists of GPR55 and GPR119 in multiple low-dose streptozotocin-induced diabetic and incretin-receptor-knockout mice
Source: Diabetologia. 2016 Sep 27;59(12):2674–85. doi: 10.1007/s00125-016-4108-z (PMC6518089; doi:10.1007/s00125-016-4108-z)
Supplement: Supplementary file 1 — (PDF 162 kb) [file 125_2016_4108_MOESM1_ESM.pdf]

Electronic Supplementary Material

Methods

Abn-CBD, CBD and AS-1269594 were purchased from Tocris Bioscience (Bristol, UK). Native GIP (1-42), GLP-1 (7-36) and Exendin (9-39) were purchased from GL Biochem (Shanghai, China). Goat anti-GPR119 polyclonal antibody (K-16; 1:100) was purchased from Santa Cruz Biotechnology (CA, USA). Guinea pig anti-insulin (1:500), guinea pig anti-glucagon (1:500) and rabbit polyclonal anti-Ki67 (1:200) were purchased from Abcam (Cambridge, UK). Rabbit anti-GPR55 polyclonal antibody (1:100) was purchased from Cambridge Bioscience Ltd (Cambridge, UK). A glucagon ELISA kit (chemiluminescent) (EZGLU-30K) and GLP-1 kit (EGLP-35K) were obtained from Millipore (MA, USA) and streptozotocin was purchased from Sigma-Aldrich (Poole, UK).

Animals

Experimental animals were individually housed in an air-conditioned room at 22 ± 2˚C with 12 hr light: 12 hr darkness cycles. Drinking water and standard rodent maintenance diet (10% fat, 30% protein and 60% carbohydrate; Trouw Nutrition, Cheshire, UK) were supplied *ad libitum.*

Chronic administration of Abn-CBD and AS-1269574 in streptozotocin-induced diabetic mice

Daily oral administration of Abn-CBD and AS-1265974 (both at 0.1µmol/kg body weight) [9, 21] or saline vehicle (0.9% w/v NaCl) were utilised in a long term study (28 days) examining their effects on multiple low dose streptozotocin-induced diabetic NIH Swiss mice. No adverse effects were noted with any of the treatment regimens. To induce diabetes, 4 hr fasted NIH Swiss mice received 5 consecutive daily intraperitoneal streptozotocin injections (40mg/kg body weight, 0.1M sodium citrate, pH 4.5). In order to confirm diabetes an oral glucose

tolerance test (OGTT) was performed 14 days after the first streptozotocin injection (day 0) on 18 hr fasted NIH Swiss mice. Food intake, fluid intake, body weight, non-fasted plasma glucose and insulin concentrations were monitored every 2 to 4 days as indicated in the Figures. At the end of the study, glucose tolerance (18mmol/kg body weight) and insulin sensitivity (25U/kg body weight) were assessed. Dual energy X-ray absorption (DEXA) scanning was performed on all carcasses, after prior calibration and quality control with the aluminium/lucite phantom (0.069 g/cm^2, 12.0% fat) using a PIXImus system (software version 1.4x).

Acute in-vivo effects of Abn-CBD and AS-1269574 in incretin receptor knockout mice

Incretin receptor knockout was confirmed by lack of effect of intraperitoneal injection of native GIP (1-42) or GLP-1 (7-36) (both at 25nmol/kg body weight) in combination with glucose administered orally (18mmol/kg body weight).

Histology

Pancreatic tissues were removed at 28 days for immunohistochemistry or measurement of pancreatic insulin content by radioimmunoassay after extraction with acid ethanol (HCl 1.5% (v/v), ethanol 75% (v/v), H_2O 23.5% (v/v)) [24]. Pancreatic tissue was fixed in 4% PFA/PBS, embedded in paraffin wax, sectioned at 8µm and morphometric analysis was performed on every tenth section throughout each pancreas. Sections were mounted onto polylysine-coated slides and dried on a hot plate. Pancreatic sections were dewaxed and antigen retrieval performed by incubation in sodium citrate (50mmol/l) at 90˚C for 20 min. Slides were incubated overnight at 4˚C with guinea pig anti-insulin (1:500), guinea pig anti-glucagon (1:500), goat anti-GPR119 (1:100), or rabbit anti-GPR55 (1:100). Rabbit anti-Ki67 was incubated at 37˚C for 2 hr. After washing in PBS, sections were incubated with anti-guinea pig IgG Alexa Fluor 488nm fluorescein; donkey anti-goat or anti-rabbit Alexa 594nm IgG (1:400; Molecular Probes (Life Technologies Ltd, Paisley, UK) for 45 min at 37˚C and DAPI

nuclear stain for 15 min at 37˚C. Finally, slides were washed in PBS, mounted and analysed using a BX51 Olympus microscope equipped with an Olympus XM10 digital camera.

Biochemical analysis

Blood samples were obtained by cut tip from tail vein of conscious mice at the time points indicated in the Figures and plasma was separated by centrifugation at 13,000 x *g* for 3 min at 4˚C. Plasma glucose was determined using an Analox GM9 glucose analyser (Analox, London, UK) and insulin measured by radioimmunoassay [25]. GLP-1 was determined using specific enzyme linked immunoassay (GLP-1 ELISA, Millipore, MA, USA). Plasma triacylglycerol and cholesterol levels were measured using a Hitachi Automated Analyser 912 (Mannheim, Germany) and glucagon ELISA assay kit performed as per manufacturers' instructions (Millipore, MA, USA).
